# West Nile Virus in Farmed Alligators

**DOI:** 10.3201/eid0907.030085

**Published:** 2003-07

**Authors:** Debra L. Miller, Michael J. Mauel, Charles Baldwin, Gary Burtle, Dallas Ingram, Murray E. Hines, Kendal S. Frazier

**Affiliations:** * University of Georgia, Tifton, Georgia, USA

**Keywords:** West Nile virus, alligators, RT-PCR, Flavivirus, food contamination, research

## Abstract

Seven alligators were submitted to the Tifton Veterinary Diagnostic and Investigational Laboratory for necropsy during two epizootics in the fall of 2001 and 2002. The alligators were raised in temperature-controlled buildings and fed a diet of horsemeat supplemented with vitamins and minerals. Histologic findings in the juvenile alligators were multiorgan necrosis, heterophilic granulomas, and heterophilic perivasculitis and were most indicative of septicemia or bacteremia. Histologic findings in a hatchling alligator were random foci of necrosis in multiple organs and mononuclear perivascular encephalitis, indicative of a viral cause. West Nile virus was isolated from submissions in 2002. Reverse transcription-polymerase chain reaction (RT-PCR) results on all submitted case samples were positive for West Nile virus for one of four cases associated with the 2001 epizootic and three of three cases associated with the 2002 epizootic. RT-PCR analysis was positive for West Nile virus in the horsemeat collected during the 2002 outbreak but negative in the horsemeat collected after the outbreak.

West Nile virus (WNV) has been reported in a variety of species but primarily endotherms. Arboviruses have been reported to affect ectotherms, and in some cases ectotherms are thought to serve as a reservoir (*1*–*4*). The mode of transmission of the arbovirus to ectotherms has often been presumed to be through ingestion or a bite from the insect carrier (*5*).

During the fall of 2001 and 2002, two epizootics occurred among captive alligators on a south Georgia alligator farm that houses over 10,000 animals. Approximately 250 alligators died between November and December 2001, and >1,000 alligators died in 2002. These epizootics tended to occur approximately 2 weeks after the first abrupt drop in ambient temperature, which occurred both years in mid-October and was characterized by minimum temperatures between 0°C and 8°C and maximum temperatures between 10°C and 18°C for a period of 1 to 3 days.

## Methods

### Animals and Housing

Animals were housed in six barns that were divided into 10 pens; each pen contained approximately 100–200 alligators. The nursery animals are obtained either as eggs from Florida or as hatchlings from onsite breeders.

All pens are cleaned in the morning starting at 6 a.m. An automatic flushing system is used to drain the pens, flush them, and fill them with clean water. Well water is chlorinated with an automated system that injects chloride gas into the water. The water is then piped into a central collecting area and heated. The water temperature is maintained at 32.2°C year-round, and the buildings are kept dark to reduce environmental stress on the animals. The reduced stress and warm environment allow continued growth (i.e., growth of >1 m per year rather than 0.30 m per year).

Alligators are fed in the mid- to late afternoon. The diet consists of 95% ground raw horsemeat (obtained frozen from a source in Pennsylvania) to which vitamins and minerals are added in a pelleted alligator diet carrier. The ingredients are thoroughly mixed in a large commercial mixer. The source of the horsemeat has remained constant since 1985. The source of the vitamins and minerals has varied, based upon availability.

The breeding population is maintained in a separate fenced enclosure on the premises. This enclosure is a native swampland and therefore subjected to ambient weather conditions. A rookery was recently established in the breeding area by native birds. Attempts to depopulate the rookery (using U.S. Department of Agriculture–approved methods) have been unsuccessful. The alligators eat fledglings and older birds that fall from the nests and branches or otherwise get within reach. Alligators do not nest under the rookery. No mosquito control is practiced on the farm.

### Tissue Collection

Animals were seen moribund or dead upon arrival at the laboratory. Blood was collected from the occipital sinus or caudal vein of live animals. Gross observations were made, and the animals were humanely euthanized. Tissues were collected from the eye, thyroid gland, lymph node, lung, heart, brain, spinal cord, kidney, liver, spleen, pancreas, adrenal gland, gallbladder, tonsil, trachea, stomach, intestines, and reproductive tract. Fresh tissue specimens were submitted for virus isolation, reverse transcription-polymerase chain reaction (RT-PCR), and bacterial culture. Tissues were also collected in 10% buffered formalin, processed, and embedded in paraffin. Five-micrometer-thick sections were stained with hematoxylin and eosin and viewed by light microscopy. Tissues opportunistically collected from an adult clinically normal, free-ranging alligator served as a control.

Multiple aliquots (totaling 1 g) of the ground raw horsemeat (without additives) that was being fed during the 2002 epizootic (October and November) were collected and processed for RT-PCR. Subsequent aliquots from postepizootic horsemeat shipments (in December and January) were similarly processed.

### Virus Isolation

A 10% homogenate in Earle’s minimal essential media (MEM) containing gentamicin was made of each specimen. The homogenate was centrifuged for 10 min at 2,000 RPM and 4°C. The supernatent was filtered and spread onto a preformed monolayer of Vero cells. In 2001, fathead minnow (FHM), white sturgeon skin (WSS), epithelioma papillosum caprini (EPC), and channel catfish ovary (CCO) cells were used instead of the Vero cells. Inoculated cells were incubated in a 5% CO_2_ atmosphere at 37°C. Cells were examined each day for viral cytopathic effect (CPE). If no CPE was observed, aliquots of the first passage were transferred to a second preformed monolayer of Vero cells (FHM, WSS, EPC, and CCO cells in 2001) on day 7. If no CPE was observed after a second 7 days of passage, the culture was considered negative. Monolayers demonstrating viral CPE were passaged to chambered slides. The slides were fixed in cold methanol, and a West Nile fluorescent-antibody test was conducted to confirm the isolate.

### Fluorescent-Antibody Testing

Mouse anti–WNV-specific polyclonal antibody (Centers for Disease Control and Prevention [CDC], Division of Vector-Borne Infectious Diseases, Fort Collins, CO) was applied to the chamber and the slide incubated in 5% CO_2_ at 37°C for 30 min. The slide was rinsed two times for 5 min in a sodium carbonate/bicarbonate buffer (pH 9.3). The slide was then air-dried, followed by an anti-mouse fluorescein-conjugated antibody, and incubated as before for 30 min. The slide was washed twice in carbonate buffer, followed by 5 min in 0.5% Evans blue counter stain. Slides were dipped in distilled water, and a glycerin/water mounting media and coverslip was added. Slides were examined with a fluorescent microscope. All isolates were tested for WNV. All isolates were also tested for Eastern equine encephalomyelitis virus (EEEV) by using a similar protocol. We tested for EEEV because of its known prevalence within the geographic area. The EEEV-specific monoclonal antibody (CDC, Atlanta, GA) was prepared against the New Jersey 1960 strain of EEEV.

### RNA Extraction

RNA was extracted from various specimens (fresh tissue, virus isolation homogenate or cell culture lysate, and formalin-fixed paraffin-embedded tissue). For extraction from fresh specimens, approximately 1 g of tissue was placed in a whirlpack bag and homogenized by using a Stomacher Lab Blender 80 (Tekmar Co., Cincinnati, OH) with three times the tissue volume of phosphate-buffered saline (PBS). Three milliliters of the tissue homogenate was processed with a Rneasy Midi kit (QIAGEN, Inc., Valencia, CA) per manufacturer’s directions. If a virus isolation homogenate or cell culture lysate in Earle’s MEM was used, approximately 4 mL of the homogenate or lysate was washed with 5 mL of PBS, the supernatant removed, and the pellet processed with the Rneasy Midi kit. For paraffin sections, several 5-µm sections from paraffin blocks were cut and deparaffinized with xylene. The xylene was removed, and samples were washed two times with 100% ethanol for 10 min, once with 95% and once with 70% ethanol. Samples were incubated overnight at 56°C in 80 µL of proteinase K with 5 mL of Buffer RLT from the Rneasy Midi kit and then processed per manufacturer’s directions.

### RT-PCR

RT-PCR for WNV was performed on the tissues according to the procedure described by Kuno (*6*) and using the RT-nested primer sets described by Johnson et al. (*7*). In brief, a RT-PCR mixture was prepared by using the outside primer set (P1401 – ACCAACTACTGTGGAGTC and P1845 – TTCCATCTTCACTCTACACT) to amplify a 445-bp product. Forty microliters of the RT-PCR mixture and 10 µL of sample were dispensed into a 0.2-mL thin wall PCR tube, and 10 µL of Rnase-free water was added for a final volume of 50 µL. With the use of a model PTC-200 thermal cycler (MJ Research, Inc., Waltham, Massachusetts), cycling conditions for the RT-PCR were as follows: 53°C for 30 min, followed by 40 cycles of 94°C for 1 min, 53°C for 1 min, 72°C for 1 min, and then held at 4°C. Ten microliters of RT-PCR first-round product was used for the nested PCR (nPCR). The nPCR mixture was prepared by using 40 µL of PCR mixture (now with the inside primer set [P1485 – GCCTTCATACACACTAAAG and P1732 – CCAATGCTATCACAGACT]) to amplify a 248-bp product. The cycling conditions for the nPCR were as described above, but the first ramp was omitted (53°C for 30 min). A 10-µL aliquot of each reaction with 1 µL of loading buffer added was loaded onto a 1.5% agarose gel in Tris-borate-EDTA (TBE) buffer and run at 70 V for approximately 1.5 h.

This protocol was repeated on all samples with primer sets for EEEV and St. Louis encephalitis virus (SLEV). For the 262-bp EEEV genomic fragment, an outer set of forward (P4 (EEE-4) - CTAGTTGAGCACAAACACCGCA) and reverse (P7 (cEEE-7) - CACTTGCAAGGTGTCGTCTGCCCTC) primers, followed by a nested set of forward (P5 (EEE-5) - AAGTGATGCAAATCCAACTCGAC) and reverse (P6 (cEEE-6) - GGAGCCACACGGATGTGACACAA) primers, was used (*8*). The RT-PCR mixture was similar to that described by Kuno (6). The thermal cycling parameters varied from those of WNV as follows: 94°C for 90 s followed by 30 cycles of 94°C for 20 s, 65°C for 35 s, 72°C for 17 s, and then a final elongation step of 72°C for 4 min. A single RT-PCR procedure was used for SLEV. The 393-bp genomic fragment was generated by using forward (SLE727 – GTAGCCGACGGTCAATCTCTGTGC) and reverse (SLE119c - ACTCGGTAGCCTCCATCTTCATCA) primers and using parameters as for WNV (*9*).

### Bacterial Culture

Swabs of individual tissues were streaked onto 5% bovine blood agar (BBA), Wilkins-Chalgren anaerobe agar, mycoplasma agar, Lowenstein-Jensen agar slant, and Hektoen Enteric agar (HE) agar (intestines only). Blood was inoculated into thioglycolate broth and streaked onto BBA. Inoculated media were incubated at 30°C with duplicate blood agar plates incubated in the presence or absence of 5% CO_2_, with the exception of the anaerobic cultures, which were incubated at 37°C. The thioglycolate broth was subcultured onto BBA after 24 h. Plates were examined each day for growth and subcultured onto BBA as needed. Bacterial colonies selected from pure cultures were Gram stained. Cultures were injected into Sensititre (Trek Diagnostic Systems, Westlake, OH) gram-negative AP80 or gram-positive AP90 autoidentification plates and the antibiotic sensitivity plate CMVIECOF and allowed to incubate for 18 h at 37°C before automated reading of the reactions per the manufacturer’s directions. Any isolates that failed to be identified by the Sensititre system were identified by using the RapID NF Plus System (Remel, Norcross, GA) or the API20E system (API Analytab Products, Plainview, NY).

## Results

### Clinical Findings

The affected alligators appeared to “star gaze” in the water just before death, suggesting neurologic lesions (*10*). Alligators sometimes became stranded in the dry part of the pen with loss of leg control and neck spasms. No long-term signs of stress were noted, and most animals were eating well until a few days before death. The hatchlings (approximately 30-cm long at the time of the epizootic) and juveniles (1–2 m long) seemed to be more severely affected.

A specific pattern of transmission was not noted in 2001. However, in 2002, the alligator deaths initially occurred in one building and spread throughout the building in the opposite direction from that taken to feed and clean the animals. At least one interruption of chlorine addition to flush water occurred before the 2002 epizootic. Deaths were not incurred in the breeding colony, and no deaths were reported in birds that inhabited the rookery.

### Gross Findings

#### 2001

Both Florida and Georgia stock animals were affected, but, in general, the Florida stock was affected first. Initially, three juvenile alligators were sent for necropsy during the 2001 epizootic. In general, the alligators were in good to excellent body condition. One alligator had approximately 25 mL of serosanguinous fluid in the pericardial sac and 50 mL yellow serous fluid in the peritoneal cavity. Two of the three had yellow-tan, caseous necrosis of the palatine tonsils and multiple caseous yellow-tan plaques, 2- to 10-mm in diameter, on the mucosal surfaces of the esophagus, corpus, and pars pylorica. Only scant ingesta were noted throughout the gastrointestinal (GI) tract, and the intestinal mucosa was hemorrhagic in rare instances. The liver and spleen of one alligator had multiple 1- to 3-mm tan foci scattered throughout the parenchyma. One alligator was in poor to moderate body condition and had scattered bronchiectasis, no ingesta throughout the GI tract, and mild multifocal serous atrophy of fat. No other gross lesions were noted.

Approximately 2 months after the 2001 epizootic began, another juvenile, live alligator was submitted to our laboratory. The gross lesions were similar to those described above but with numerous 1- to 3-mm tan foci in the parenchyma of the liver, spleen, and kidneys.

#### 2002

Three alligators were examined from the fall 2002 epizootic, two juveniles and a hatchling. The two juveniles had lesions similar to those described in the previous year. The liver and kidneys of the hatchling were pale and mottled tan/brown. Ingesta were scant throughout the GI tract. The free-ranging alligator was in excellent body condition. No significant gross changes were noted in its tissues.

### Light Microscopic Findings

#### 2001

**Figure 1:**
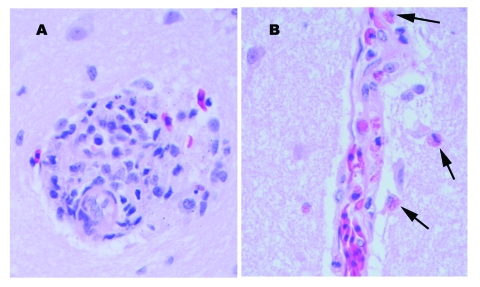
Perivascular changes observed within the brain of alligators infected with West Nile virus (400x). A. Perivascular infiltrates were composed of primarily lymphocytes, plasma cells, and macrophages in the hatchling alligator. B. Perivascular infiltrates were composed of primarily heterophils (arrows) in juvenile alligators.

Tissues of the alligators from the 2001 epizootic were examined and were similar in two of the three alligators. In the brain, rare glial nodules that contained occasional heterophils were present (Figure 1). The spleen was congested with moderate diffuse reticuloendothelial hyperplasia and moderate numbers of heterophils. The tonsil had severe multifocal coalescing areas of caseous necrosis and heterophilic inflammation with reactive lymphoid follicular hyperplasia. In the esophagus, a focally extensive, mixed ulcerative, and proliferative lesion was present; it had a marked mixed but predominantly mononuclear inflammation, colonies of bacteria, and extensive fibrin deposition. In the liver, multifocal lymphoplasmacytic aggregates and heterophilic granulomas were present, consisting of caseous necrotic foci with degenerate heterophils surrounded by an outer layer of macrophages, lymphocytes, and heterophils. The lungs were congested with mild diffuse or patchy lymphoplasmacytic and heterophilic interstitial infiltrates. The kidney had multifocal heterophilic granulomas. The pars pylorica region of the stomach had multifocal mucosal abscesses and moderate diffuse lymphoplasmacytic and heterophilic infiltrates of the lamina propria. The small intestine had moderate, diffuse mucosal and submucosal infiltrates of lymphocytes, heterophils, and plasma cells and multifocal areas of acute necrosis associated with bacteria. The remaining tissues appeared within normal limits. Special stains for fungi and acid-fast bacteria were negative. A population of primarily gram-negative and fewer gram-positive bacteria was observed in the heterophilic granulomas.

The third alligator had primarily pulmonary changes. The airways contained moderate numbers of heterophils, occasional mucous plugs with degenerate inflammatory cells, and scattered bacterial colonies. The remaining tissues were as described for the first two alligators.

Tissues from the alligator seen 2 months after the epizootic had similar findings to those of the first two alligators with the addition of rare, small caseating granulomas within the lungs. The granulomas contained numerous large macrophages and multinucleated cells. Acid-fast stains demonstrated low numbers of slender, beaded, acid-fast positive bacilli consistent with mycobacteria.

#### 2002

Multiple tissues from the two juvenile alligators from the 2002 epizootic were examined. The tissue changes were similar to those described for the 2001 epizootic except that the inflammatory component was primarily heterophils. The meninges within the brain and all spinal cord sections except those from the sacral spinal cord had stasis of heterophils within the blood vessels and perivascular infiltration of mild numbers of heterophils (Figure 1). One alligator had a small focus of macrophages and heterophils noted within the endocardium.

Multiple tissues were examined from the hatchling alligator, and lesions differed from the previous submissions on the basis of cellular composition of the inflammatory cell infiltrates. Lymphoplasmacytic perivascular cuffs were present throughout the brain and meninges (Figure 1). Rarely, heterophils were admixed within the cuffs. Similar changes were not seen within the spinal cord. Random foci of necrosis were seen within the liver, pancreas, and tonsil. Mild to moderate perivascular infiltrates of lymphocytes, plasma cells, and heterophils were seen within the kidney and heart, and similar but fewer numbers of infiltrates were seen within the pulmonary interstitium. The heart had multiple, random foci of patchy vacuolar degeneration of the myocytes and random aggregates of lymphocytes, plasma cells and heterophils. Mild numbers of mixed inflammatory cells were seen within the intestinal lamina propria. The remaining tissues were unremarkable. Major pathologic changes were not observed by light microscopy in the tissues from the free-ranging alligator.

### Virus Isolation/RT-PCR

**Figure 2:**
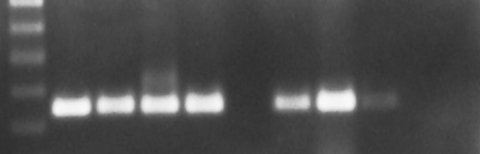
West Nile virus (WNV) reverse transcription-polymerase chain reaction results from epizootic die-offs in farm-raised alligators. The expected amplicon is 248 bp. Lane 1, a 100-bp molecular weight ladder. Lane 2, the positive WNV control. Lane 3, fresh tissue samples from a juvenile alligator in the 2002 epizootic. Lane 4, virus isolation cell homogenate from a juvenile alligator in the 2002 epizootic. Lane 5, horsemeat that was being fed to alligators during the 2002 epizootic. Lane 6, initial postepizootic horsemeat shipment. Lanes 7, 8, and 9, formalin-fixed, paraffin-embedded tissues of juvenile alligators in 2001 and 2002. Lane 10, fresh tissue from a wild alligator. Lane 11, negative WNV control.

Virus isolation was negative for all animals from the 2001 epizootic. WNV was isolated from tissues from all animals in the 2002 epizootic. Additionally, all animals from the 2002 epizootic and one animal from the 2001 epizootic were positive for WNV by RT-PCR from fresh or formalin-fixed, paraffin-embedded tissues (Figure 2). In general, liver was the most likely tissue to yield positive results. Positive results were not obtained from any of the tissues from the free-ranging alligator. All tissues tested negative by RT-PCR for EEEV and SLEV. Retrospective attempts to culture WNV at both 37°C and room temperature on FHM, CCO, EPC, and WWS cells were negative.

Aliquots from the horsemeat that was being fed during the 2002 epizootic tested positive for WNV by RT-PCR (Figure 2). Aliquots of the horsemeat from two postepizootic shipments were negative for WNV by RT-PCR.

### Bacterial Culture

*Aeromonas sobria* and *Edwardsiella tarda* were consistently cultured from the intestines. These organisms and occasionally others (*Escherichia coli*, *Pseudomonas* *fluorescens*, α- and β-hemolytic *Streptococcus*) were isolated from various tissues (liver, lung, and kidney) from the alligators dying during the 2001 epizootics and the juveniles from the 2002 epizootics. *Alcaligenes* spp. were isolated from a tonsil swab in one of the animals in 2001. *Salmonella* Group D was isolated from the intestines of the hatchling alligator submitted in 2002.

## Discussion

The histologic findings from the hatchling alligator were most suggestive of a viral etiology, whereas those of the older alligators were most suggestive of a primary bacterial cause. Given that both the RT-PCR and virus isolation were positive for WNV, that virus is suspected to be the underlying cause of both epizootics. Contaminated horsemeat is the presumed source of the outbreak. We speculate that the WNV infection led to the alligators’ immune systems’ becoming immunocompromised, which resulted in the animals being more susceptible to various environmental stressors and subsequent invasion by opportunistic pathogens. Failure to isolate virus from the alligators in 2001 may have been due to the inability of the virus to propagate in the four cell lines used (FHM, CCO, EPC, and WWS cells), as determined by retrospective culture attempts, rather than absence of virus.

Two important points to examine further are time of year and age of affected animals. Both epizootics occurred in the late fall to early winter. Although the epizootics appeared to be correlated with the first abrupt drop in environmental temperature, this finding was likely coincidental, especially given that the animals were housed in environmentally controlled barns. The most likely factor in the time of year is correlation with the occurrence of WNV infection in horses. Historically, horses become infected with WNV during the mosquito season (summer through early fall). Undiagnosed WNV-infected animals sold for food would most likely end up in the food supply during the late summer and early fall months. As was found in this study, deaths traced to consumption of contaminated food would taper off in late fall or early winter as the food supply was less likely to contain virus. Furthermore, all animals have equal potential for viral exposure through consumption because individual packages of horsemeat are combined before mixing with the vitamin supplements and being divided between all barns. In general, reptiles achieve immunocompetence at an early age (often in a matter of days), but this immunocompetence may be temperature dependent until the animals are several months of age (*11*). This fact may partially explain why the hatchling alligators tended to die from the viral infection, whereas the juveniles tended to die from infections caused by secondary invaders.

Extrinsic stressors may have increased certain animals’ susceptibility to the virus or opportunistic pathogens. For example, the pens where the epizootics originated tended to be the first to be washed out at 6 a.m., the coolest time of the day. During the first abrupt drop in environmental temperature, the first wash water was possibly cooler because of colder water in the line between the boiler and the pens. This cold stressor would serve as a shock to the animals’ systems. During the 2002 outbreak, an additional stress was internal construction, undertaken 2 weeks before the epizootic within the initially affected building. The environmental (temperature and darkness) control of the building was maintained during this time, but silence was not maintained. Additionally, sanitation-related stress may have occurred during periods of intermittent flushing, such as over weekends and during pen renovation activities.

Whether brood stock source had an affect on the susceptibility of the animals is not clear. Although Florida stock animals were those initially affected, this finding was likely coincidental because of their location in the pens. The pens that were more exposed to external stressors contained Florida animals. Additionally, most animals in the production unit are from Florida brood stock.

Several management recommendations were suggested to the producer. The primary recommendation was to stop feeding horsemeat and switch to another food source such as beef or fish. We also recommended that the water temperature be reduced to 29.4°C in an attempt to reduce the stress of rapid growth and perhaps produce an environment less conducive for viremia. To date, neither of these recommendations has been implemented, but subsequent horsemeat shipments have tested negative. Future investigation will include the testing of the eggs from the brood stock, clinically healthy animals, rookery birds, and free-ranging alligators to explore the epidemiology of this virus in ectotherms.
